# KPNA2 promotes metabolic reprogramming in glioblastomas by regulation of c-myc

**DOI:** 10.1186/s13046-018-0861-9

**Published:** 2018-08-16

**Authors:** Jie Li, Qian Liu, Zihao Liu, Qian Xia, Zihao Zhang, Rui Zhang, Taihong Gao, Guangyan Gu, Yanan Wang, Dan Wang, Xiuyang Chen, Yihang Yang, Dong He, Tao Xin

**Affiliations:** 1 Department of Neurosurgery, Shandong Provincial Hospital Affiliated to Shandong University, Jinan, 250021 Shandong China; 2 Department of Histology and Embryology, Shandong University Cheeloo College of Medicine, Jinan, 250012 Shandong China; 3 Jiangxi Medical College, Nanchang University, Nanchang, 330006 Jiangxi China; 4 Jiangxi Provincial People’s Hospital, Nanchang, 330000 Jiangxi China

**Keywords:** KPNA2, Warburg effect, c-myc, Glioma, E2F1

## Abstract

**Background:**

Cancer cells maintain energy metabolism mainly by glycolysis, even under sufficient oxygen conditions. It gives cancer cells better growth advantages under complicated internal environment. KPNA2 is a novel oncogene that has received much attention in recent years, but the exact mechanisms of KPNA2 in tumorigenesis and progression are largely unknown. Especially its potential roles in the metabolic transformation of tumors still remain to be explored.

**Methods:**

The expressions of KPNA2 in glioblastoma and normal human brain samples were analyzed by immunohistochemical analysis. The activities of key enzymes in glycolysis, the production of lactate acid and glucose uptake were investigated by colorimetry. GLUT-1 expression was measured by flow cytometry. CCK8 was used to examine the cell viability in vitro, and the xenograft models in nude mice were established to explore the roles of KPNA2 in vivo. In addition, Co-IP, subcellular fractionation, western blot, immunofluorescence and luciferase assay were used to investigate the internal connection between KPNA2, c-myc and E2F1.

**Results:**

In the present study, we found that KPNA2 was highly expressed in the glioma compared to the normal brain tissues. Level of KPNA2 was an independent predictor of prognosis in the glioma patients. Knockdown of KPNA2 in the glioblastoma cell lines U87 and U251 decreased deoxyglucose uptake, activities of the key glycolytic enzymes and lactate production. The level of oxidative phosphorylation (OXPHOS) was moderately decreased. Additioanlly, tumor proliferation and invasiveness were concomitantly downregulated. We have identified c-myc as a potential mediator of KPNA2. Aberrant expression of KPNA2 significantly changed the subcellular distribution of c-myc as well as its expression level. E2F1, another key cargo protein of KPNA2, was further identified to play a potential role in regulating the transcription of c-myc by KPNA2.

**Conclusions:**

Our findings suggested that KPNA2, a potential tumor oncogene, performs its function in part via regulating cellular metabolism through c-myc signaling axis. It would provide a possible explanation for Warburg effect and thus offer a new perspective to the roles of KPNA2 in gliomagenesis.

**Electronic supplementary material:**

The online version of this article (10.1186/s13046-018-0861-9) contains supplementary material, which is available to authorized users.

## Background

According to the American Cancer Society and Annals of Translational Medicine, central nervous system invasive cancers account for 3% of all cancers, and the morbidity and mortality have increased year by year [1, 2], while the vast majority of which are gliomas. Due to the infiltrative nature of gliomas, although the current treatments, including surgical resection, adjuvant radiotherapy and chemotherapy have made a wealth of research results, their prognosis is extremely poor and death occurs inevitably from either recurrence or disease progression [3]. Patients with glioblastoma multiforme (GBM), a type IV glioma based on pathological criteria, for instance, have a median survival time of only about 15 months [4].

Cancer cells mainly depend on glycolysis for energy metabolism, even when there is sufficient oxygen. This is the core content of Warburg effect. It helps cancer cells survival under fluctuating oxygen tension microenvironment, which is lethal for normal cells [5]. It was reported that in glioblastomas, glycolytic metabolism is 3 times higher than normal brain tissues [6], which can be regulated by several oncogenes and tumor suppressor genes, such as c-MYC and HIF-1 [7]. Although so much work has been performed on the transformation between glycolysis and oxidative phosphorylation of gliomas, the mechanism of Warburg effect is still unclear.

Dysfunction of nucleocytoplasmic transport is commonly observed in many malignant biological behaviors, including gilomas [8]. Nucleocytoplasmic transport occurs when molecules want to get through the Nuclear Pore Complexes (NPCs) in the nuclear membrane. Karyopherins are responsible for the shuttling of macromolecules larger than about 40 kDa. The karyopherin family includes import (importins) and export factors (exportins). More than 20 members of the karyopherin family have been described [9, 10]. Importin α serves as an adaptor that links the nuclear localization signal(NLS) containing cargo proteins to the NPCs, and when the NLS recognized, importin β docks the ternary complex at the NPC and facilitates the translocation of the cargo proteins into the nucleus [11]. Karyopherin α2 (KPNA2, also known as importinα-1 or RAG cohort 1) is one of the seven members of karyopherin α family, which is made of 529 amino acids and is about 58 kDa [12–15]. Many studies have linked KPNA2 with malignances: Dahl et al. reported that high KPNA2 level was correlated with poor prognosis and high recurrence rates in breast cancers [16]. Elevated expressions of KPNA2 were also reported in other types of malignancies, such as hepatocellular carcinoma, endometrial, prostate, colorectal and brain cancers [17–20], with an average 5–10-fold increase than in normal cells. What’s more, aberrant KPNA2 expression has been found in early lesions, such as non-invasive bladder cancer and ductal carcinoma in situ (DCIS) in the breast cancer samples. These findings evidently suggested that KPNA2 could potentially participate in the development of tumors [21]. However, even though the oncogenous effects of KPNA2 have been explored for years, the exact mechanisms of KPNA2 in tumorigenesis and progression are largely unknown. Especially its potential role in the metabolic transformation of tumors still remains to be explored.

In the present study, we found that KPNA2 was highly expressed in the glioma compared to the normal brain tissues, and the level of KPNA2 was an independent predictor of prognosis in the glioma patients. Knockdown of KPNA2 in the glioblastoma cell lines U87 and U251 decreased deoxyglucose uptake, activities of the key glycolytic enzymes and lactate production. Meanwhile, the level of oxidative phosphorylation (OXPHOS) was moderately decreased. Tumor proliferation and invasiveness were concomitantly downregulated. We have identified c-myc as a potential mediator of KPNA2. Aberrant expression of KPNA2 significantly changed the subcellular distribution of c-myc as well as its expression level. E2F1, another key cargo protein of KPNA2, was further identified to play a potential role in regulating the transcription of c-myc by KPNA2. As a result, KPNA2, a potential tumor oncogenic protein, performs its function in part via regulating cellular metabolism through c-myc signaling axis. It would provide a possible explanation for Warburg effect and thus offer a new perspective to the roles of KPNA2 in gliomagenesis.

## Methods

### Glioma samples and cell lines

A total of 105 glioblastoma samples and 8 normal human brain samples were obtained from the Department of Neurosurgery at Provincial Hospital affiliated with Shandong University. The research was approved by Research and Ethics committee at school of medicine, Shandong University, China. And all the patients chosen had provided written informed consent according to the committee’s regulations. The glioblastoma cell lines U87 and U251 were acquired from Procell Life Science &Technology Co. Ltd. (Wu Han, China). All the Cells were maintained in the DMEM medium with high glucose and sodium pyruvate (Gibco,Life Technologies, Carlsbad, CA, USA), supplied with 12% fetal bovine serum(FBS, BI, Grand Island, NY, USA.), 100 units/mL penicillin and 100 μg/mL streptomycin (Life Technologies)and were incubated at 37 °C with a 5% CO2 humidified atmosphere.

### Construction of shRNA plasmids

KPNA2 cDNA was cloned into the Lenti-OE vector (Genechem, Shanghai, China) to generate KPNA2-overexpressing lentiviral vectors. The shRNAs were cloned into GV492 vector carried by Lenti-OE (Genechem, Shanghai, China), and the knockdown targeted sequences was as follows: KPNA2, 5′ -ATTTACAGTGCCCTGGTTG-3′; c-myc, 5’-ATGTCAAGAGGCGAACACA-3′; E2F1, 5’-TAGATC CGATCCAGCTCAGTGACA-3′, scramble, 5′- TTCTCCGAACGTGTCACGT -3′. The U87 and U251 cells were infected by the lentiviruses, and incubated for 24 h.

### Western blot assay

Cells were lysed and sonicated in the buffer containing 1% Triton X-100, 10 mmol/L TrisHCl (pH 7.4) and proteases/phosphates inhibitors (Roche Diagnostics, USA), electrophoresed in 10% SDS-PAGE gel electrophoresis, and transferred to a polyvinylidene difluoride membrane (PVDF) probed with primary antibodies to KPNA2. Subsequently, the membrane was probed with horseradish–peroxidase conjugated secondary antibodies. Then the blots were detected and visualized by a chemiluminescence detection system (Pierce, Rockford, IL, USA). The primary antibodies were used: anti-KPNA2 (Abcam, Cambridge, MA, USA), anti-c-myc(Abcam, Cambridge, MA, USA), anti-histoneH3 (Boster, Pleasanton, CA, USA); anti-E2F1 (Abcam, Cambridge, MA, USA), and anti-GAPDH (Boster, Pleasanton, CA, USA).

### Immunohistochemistry (IHC)

Paraffin-embedded glioblastoma blocks were cut into 5 μm thick sections and then subjected to antibody specific for KPNA2 (Abcam, Cambridge, MA, USA) at 4 °C for 12 h, followed by secondary antibody (ZSGB. BIO, Beijing, China) applied to tissues at 37 °C for 30 min. The staining intensity was classified as four grades: 0(no expression or no positive staining), 1(1–10% positive cells or light yellow), 2(11–50% positive cells or brownish yellow), 3(51–75% positive cells or brown), 4(> 75% positive cells or dark brown).

### Quantitative real-time PCR (qRT-PCR)

Total RNA was isolated from different cell lines using Trizol reagent (Invitrogen, USA). QRT-PCR was performed using a Real-time PCR System (Applied Biosystems, USA) with a SYBR Green PCR kit (TaKaRa, Japan) according to the manufacturer’s instructions. The primer sequences were as follows:

KPNA2, 5’-ATTGCAGGTGATGGCTCAGT-3′ (forward) and 5’-CTGCTCAACAGCATCTATCG-3′ (reverse);

c-myc, 5’-GGAGCCTATTCTGCCCATTT-3′(forward) and 5’-CGAGGTCATAGTTCCTGTTGGTG-3′(reverse);

HK2, 5’-TCCGTAGTGGGAAAAAGAGAA-3′ (forward) and 5’-GACAATGTGATCAAACAGCTC-3′ (reverse);

PFK1, 5’-GGTGTACAAGCTTCTAGCTC-3′ (forward) and 5’-CAAGTTTAGAGCCACCTTGG-3′ (reverse);

PKM2, 5’-CCACTTGCTGTGCCAAATGGA-3′ (forward) and 5’-GAAGGACTTTACCTTCCAGGA-3′ (reverse);

PDK1, 5’-GAAGCAGTTCCTGGACTTCG-3′ (forward) and 5’-ACCAATTGAACGGATGGTGT-3′ (reverse);

GLS, 5’-CGACACTGGCTAATGGTGGT-3′ (forward) and 5’-TGCAGGAAGACCAACATGGAA-3′ (reverse);

SLC1A5, 5’-CCTGGATCATGTGGTACGCC’ (forward) and 5’-GAAGCGGTAGGGGTTTTTGC-3′ (reverse);

and GAPDH, 5’-GCACCGTCAAGGCTGAGAAC-3′ (forward) and 5’-TGGTGAAGACGCCAGTGGA-3′ (reverse).

### Enzymatic activities of PFK, PKM and HK

U87 and U251 cells were seeded in six-well plates at a density of 2.0 × 10^5^ per well, and cultured for 12 h before transfection. The cells were then digested by 0.08% EDTA trypsin after 48 h. The enzymatic activities of PFK, PKM and HK were detected by the respective enzyme activity kit (Jiemei Genetech, Corporation, China) according to the manufacturer’s instructions.

### Detection of lactate levels

A lactate assay kit (Jiancheng Corporation Ltd., Nanjing, China) was used to measure the lactate level in the culture medium 24 h after transfection. The lactate was oxidized by lactate dehydrogenase, and generated a product that could interact a probe to produce colors (an absorption maximum at 492 nm). Total viable cell number was used for normalization.

### Pyruvate dehydrogenase (PDH) colorimetric assay

Cells were seeded and dissolved by 100 μl ice cold PDH Assay Buffer. Mitochondria from the cultured cells were isolated using a Biovision’s Mitochondria Isolation Kit. According to the protocols of Pyruvate Dehydrogenase Activity Colorimetric Assay Kit(Biovison, San Francisco, USA), 0, 2, 4, 6, 8 and 10 μl of 1.25 mM NADH Standard were added into the wells of 96-well plate and Color densities were measured (OD 450 nm) using a microplate reader (Tecan, Infinite M200, Switzerland) for 10–60 min at 37 °C. Then plot of the NADH Standard Curve used the protein concentration as standardization.

### Colorimetric assay on glucose uptake

Cells were seeded in a 96-well plate at a density of 1000 per well and incubated for another 4 days prior to use. The assay was performed using a Glucose Uptake Colorimetric Assay Kit (Biovison, San Francisco, USA) according to the manufacturer’s protocols. The absorbance at 412 nm was measured using a microplate reader (Tecan, Infinite M200, Switzerland) at 37 °C every 5 min until the 100 pmol standard reached 1.8.

### Flow cytometry (FCM) analysis

The U87 cells were seeded in a 6-well plate at a density of 2.0 × 10^5^/ well. 48 h after transfection, the cells were digested by 0.08% trypsin and washed with ice-cold PBS for three times. After that, cells were marked with Alexa Flour®647-GLUT1 antibody (Abcam, Cambridge, USA), and the emission was measured using a FACS Calibur cytometer (Beckman coulter, KBB, CA, USA).

### Extracellular acidification and oxygen consumption rates (ECAR and OCR)

Cells were seeded in the 24-well plates and incubated for 24 h. The culture medium then was replaced with running buffer 30 min prior to the assay. The mitochondrial inhibitor oligomycin (1 mM) was added into the plates after 20 min and the uncoupler FCCP (300 mM) was added after another 20 min. The ECAR and OCR were then determined by a Seahorse XF24 extracellular Flux analyzer.

### Cell viability assay

Cell suspension was incubated in the 96-well plates at a density of 5000 cells per well and placed in a humidified incubator (37 °C, 5% CO2) for 24 h. Then 10 μL of the reageant cell-counting kit8 was added into each well of the plates (CCK-8, BestBio, Shanghai, China). Two hours later, the spectrometric absorbance was measured at 450 nm using a microplate reader (Tecan, Infinite M200, Switzerland).

### In vivo tumor formation

The glioblastoma cells (1× 10^5^) were injected into 4 week-old male athymic Nu/Nu mice (Cancer Institute, Medical Science, Chinese Academy) according to the Guide for the Care and Use of Lab Animals. The research was approved by the Animal Care and Use Committee of Shandong University. Transduced cells were subcutaneously injected into the right frontal lobes of the mice. The nude mice were anesthetized and dissected 4 weeks later. Tumor volumes were monitored and overall survival rate was analysed. The tumor volumes were determined as follows: Maximal and minimal diameter of the tumor was measured on the H&E stained sections. Tumor volume was calculated from the formula: Tumor volume = (max. diameter) × (min. diameter) 2 × 0.5 [22, 23].The expressions of PFK1, PKM2 and HK2 were detected by qRT-PCR.

### Subcellular fractionation

The cells were harvested and washed with PBS for two times, and subjected to subcellular fractionation by a Minute TM Cytoplasmic and Nuclear Fractionation kit (Invent Biotechnologies, Inc. Eden Prairi. US) referring to the manufacturer’s protocols. The efficacy of fractionation was tested by immunoblotting using anti-Histone H3 antibody (Boster, Pleasanton, CA, USA) as the nuclear protein control, while using anti-MEK1/2 antibody (Abcam, Cambridge, MA, USA) as the cytosolic protein control.

### Immunofluorescence

The glioblastoma cells were fixed in 4% paraformaldehyde for 15 min at room temperature,rinsed in 0.3% Trion X-100 for 5 min and were placed on the coated coverslips After blocked with goat serum(ZSGB. BIO, Beijing, China) for 30 min, they were incubated with the primary antibody to c-myc and E2F1 (Abcam, Cambridge, MA, USA)at 37 °Cfor 1 h. Then the coverslips were washed with PBS and stained with Alexa Fluor 594--labelled secondary antibodies followed by the staining with 4′,6-diamidino-2-phenylindole (DAPI). A fluorescent microscope was utilized at last to watch the fluorescence**.**

### Co-IP assay

Firstly, the cells were seeded, transfected and lysed with a gentle detergent, producing a mixture containing the bait–target complexes and other irrelevant proteins. Then a capture antibody together with protein A/G plus agarose was added into the mixture to bind the bait protein specifically after the antibody was crosslinked to the agarose by using the DSS crosslinker, the sample was prepared for SDS-PAGE analysis. All the procedures were referred to a Pierce Crosslink Immunoprecipitation Kit (Thermo Fisher scientific, invitrogen, German).

### Luciferase assay

The U87 and U251 cells were seeded in the 24-well plates before transfection. 48 h later, cells were lysed by PLB (1×, 100ul) and put on an shaker (QILINBEIER, China) at 100 rpm for 30 min and then placed in − 80 °C refrigerator for at least 12 h. Firefly luciferase signals and renilla luciferase (internal reference) were detected by a dual-luciferase reporter assay kit (Promega, Madison, WI, USA.) referring to the manufacture’s protocols.

### Statistical analysis

The software of GRAPH PAD prism 5.0 (Graphpad, software, Inc., LaJolla, CA, USA) was used for all the statistical analyses. A two-tailed student’s t-test was utilized to analyze the differences between two groups, while one-way analysis of variance (ANOVA) was used to compare the differences between multiple groups. Kaplan and Meier method was used to assess the overall survival (OS) or progression free survival (PFS) and the curves were analysed by log-rank. All experiments were performed for at least 3 replicates and a *p*-value < 0.05 was considered as statistically significant.

## Results

### Overexpression of KPNA2 in the glioma tissues predicted poor prognosis of patients

**Fig. 1:**
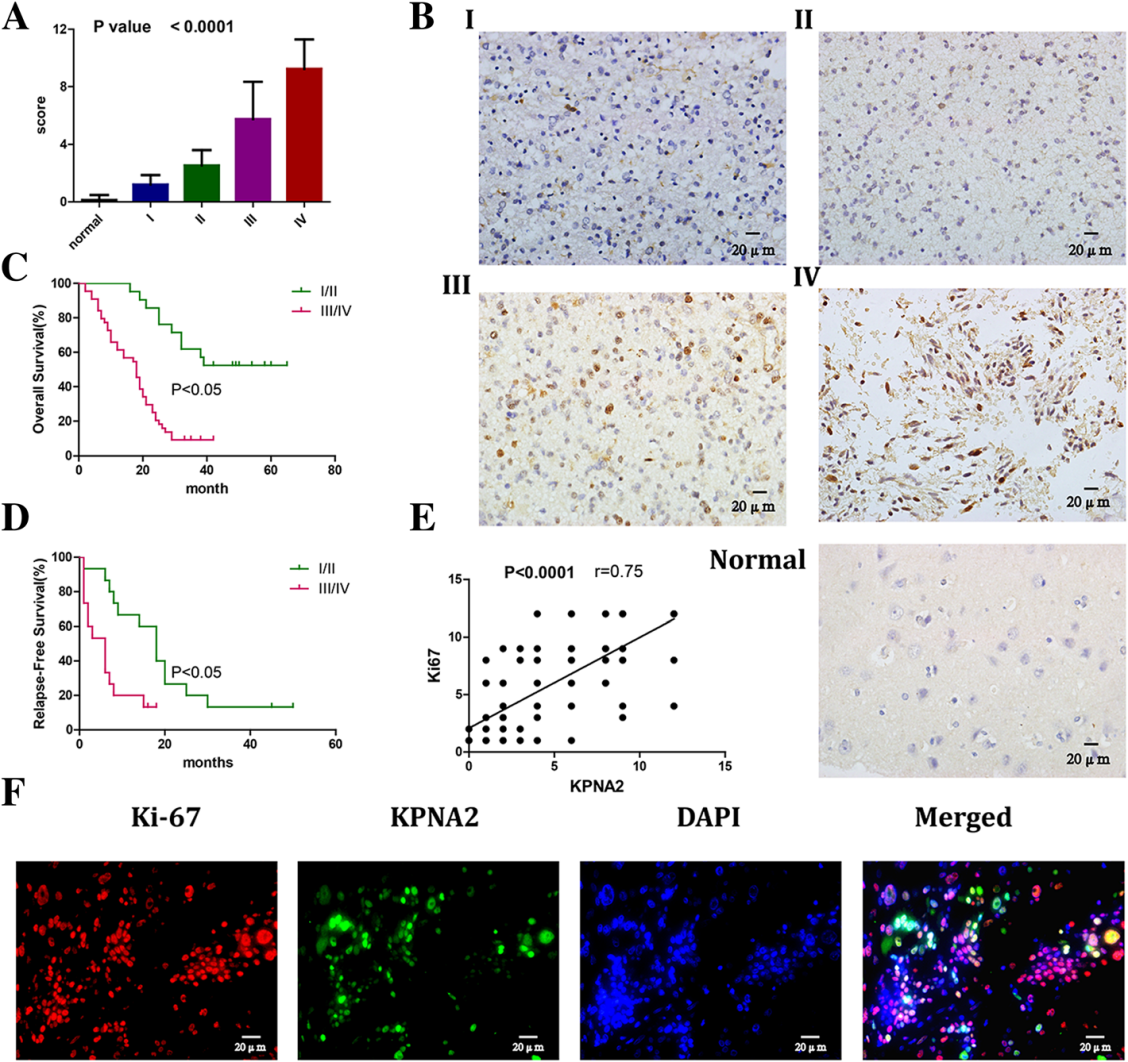
KPNA2 was overexpressed in the glioma tissues and positively correlated with poor prognosis of the glioma patients. **a** The staining of KPNA2 was scored based on the criteria in the methodology. Data were presented as mean ± s.d. from three independent experiments. *P* <  0.0001 between every two adjacent pillars. **b** Immunohistochemical (IHC) images of KPNA2 in the glioma tissues and normal brain tissues were shown (400×). Clinical glioma specimens were classified into four World Health Organization (WHO) grades (I: Pilocytic astrocytomas, II: Astrocytomas, III: Anaplastic astrocytomas, and IV: glioblastomas). **c** Overall survival and **d** relapse-free survival of the glioma patients were followed up based on WHO grades(I,II vs. III,IV). *P* <  0.05 is considered statistically significant. **e** Correlation of the expressions of KPNA2 and ki-67 in the glioma specimens was shown (*P* <  0.0001). **f** Double immunohistological staining of Ki-67 and KPNA2 in the glioblastoma specimen (400×)

To investigate the expression levels of KPNA2 in the glioma patients, 105 glioma tissues were collected, including 15 pilocytic astrocytomas(WHO grade I), 30 astrocytomas (WHO grade II), 30 anaplastic astrocytomas(WHO grade III) and 30 glioblastomas(WHO grade IV). Eight non-neoplastic brain tissues were used as the controls. Immunohistochemical (IHC) analysis was performed and the results showed that the expression of KPNA2 was increased with the grades of malignancy in gliomas (Fig. 1a, b). The survival analysis on these patients indicated that overexpression of KPNA2 was significantly associated with decreased overall survival and relapse-free survival (Fig. 1c, d). On the other hand, correlation between the expressions of KPNA2 and ki-67 were shown (Fig. 1e), further demonstrating that upregulation of KPNA2 was associated with poor prognosis of the glioma patients. What’s more, we performed double immunohistological staining of KPNA2 and Ki-67 on the glioma specimen. The results showed that the proliferative tumor cells tended to express higher amount of KPNA2. Additionally we observed that KPNA2 appeared to be predominantly situated in the nuclei of glioma cells (Fig. 1f).

We subsequently followed up these 105 glioma patients and performed hazard ratio analysis (Table 1). The results indicated that overexpression of KPNA2 was indeed an independent risk factor of gliomas in the patients.

**Table 1:** Univariate analysis of factors associated with overall survival

Variables	Hazard ratio (95%CI)	*P*-value
Age(years)	0.5579 (0.3431 to 0.9073)	0.0196
< 50		
≧50		
Tumour size(cm)	0.9748(0.5803 to 1.638)	0.0109
< 3		
≧3		
Histology WHO	0.04285 (0.02234 to 0.08218)	< 0.0001
I/II		
III/IV		
KPS	0.5097 (0.3145 to 0.8261)	0.0002
<=80		
> 80		
Chemotherapy	0.8774 (0.5482 to 1.404)	0.9565
yes		
no		
radiation therapy	0.9376 (0.5714 to 1.539)	0.3189
yes		
no		
ki-67	0.1614 (0.09453 to 0.2756)	< 0.0001
Negative		
Positive		
KPNA2	0.5861 (0.2461 to 0.9832)	< 0.0001
<8		
≥8		

### High level of KPNA2 in the glioblastoma cells enhanced glycolytic metabolism

**Fig. 2:**
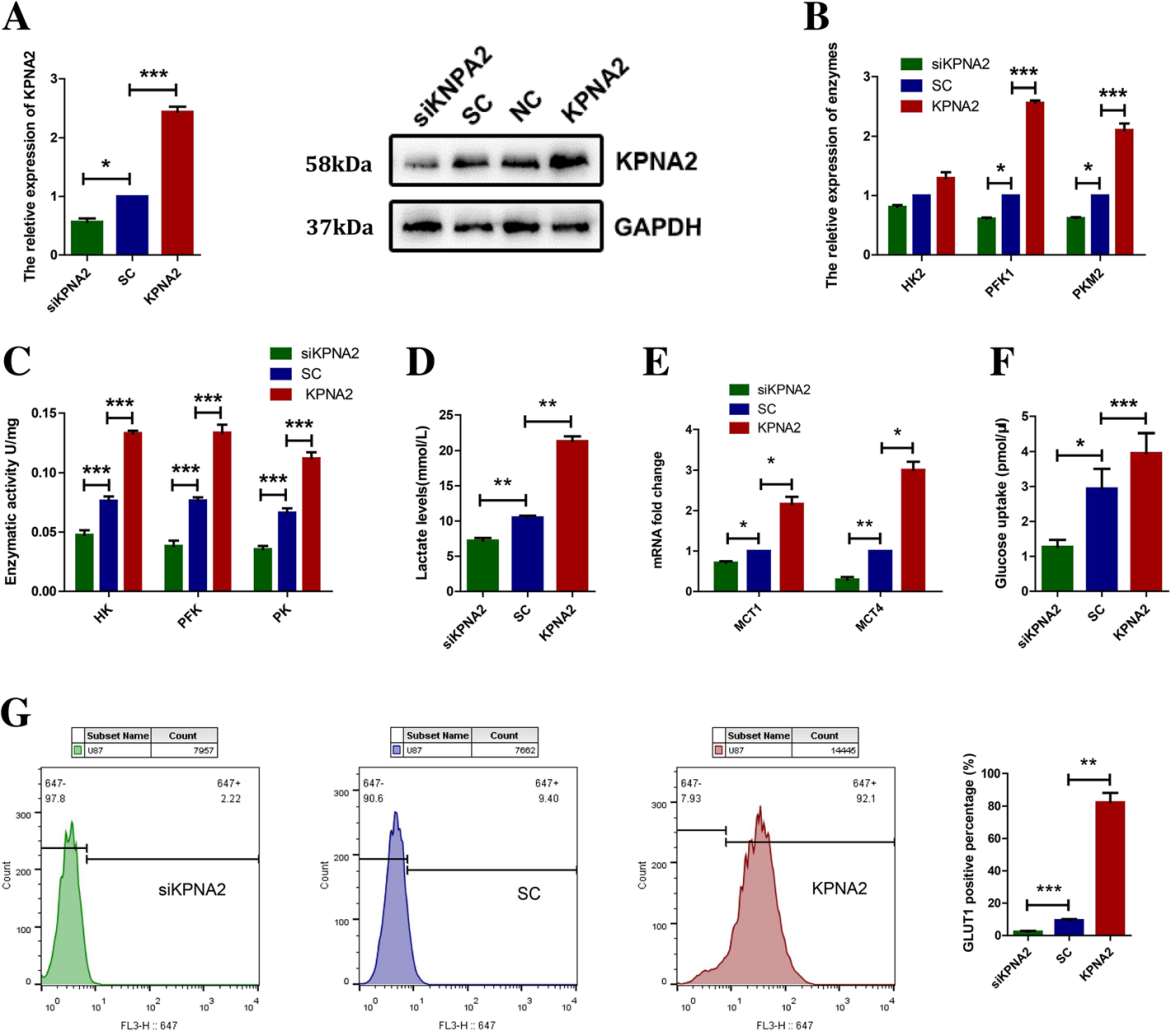
KPNA2 promoted the glycolytic metabolism in the glioblastoma cells. **a** Levels of KPNA2 were analyzed by qRT-PCR and immunoblotting in the U87 glioblastoma cells transfected with the lentiviruses expressing small hairpin RNAs of KPNA2 (shKPNA2), wild-type KPNA2 (KPNA2), or a vector with scrambled nonspecific shRNAs(SC). NC is for the original U87 cells that without any handles, GAPDH served as a loading control. **b** mRNA levels and **c** enzymatic activities of HK2, PKM2 and PFK1 were determined in the U87 cells transfected with KPNA2-shRNA(siKPNA2), scrambled shRNA(SC) or wild-type KPNA2(KPNA2). Each bar represented the mean ± s.d. from three independent experiments. *P <  0.05, ***P <  0.001. **d** Lactate production was measured in the indicated cells. Data were presented as the mean ± s.d. from three independent experiments. **P <  0.01. **e** MRNA levels of MCT1 and MCT4 in the U87 cells. Data were presented as the mean ± s.d. from three independent experiments. *P < 0.05, **P < 0.01. **f** Relative deoxyglucose uptake was measured in the indicated cells. Each bar represented the mean ± s.d. from three independent experiments. ***P < 0.001. **g** The expression of GLUT-1 was detected by flow cytometry in the indicated cells, cells were marked with Alexa Flour®647-GLUT1 antibody and the statistic analyze were followed. Data were presented as the mean ± s.d. from three independent experiments. **P < 0.01. ***P < 0.001

To investigate the role of KPNA2 that plays in the energetic metabolism of gliomas, considering the different P53 status may be an important factor in cellular metabolism. We separately transfected a lentiviral vector of KPNA2 shRNA, wild type KPNA2 or their comparative controls into the U87 (harboring wild-type P53) and U251 (harboring mutant P53) glioblastoma cell lines. The results of quantitative real-time polymerase chain reaction (qRT-PCR) and western blot indicated that KPNA2 was notably upregulated in the KPNA2 transfectants and depleted in the KPNA2-shRNA transfectants (Fig. 2a, Additional file 1: Figure S1A).

To assess the glycolytic activity of these cells, we first detected the mRNA levels and enzymatic activities of phosphofructokinase (PFK), pyruvate kinase (PKM) and hexokinase (HK), which are key enzymes in the glycolytic pathway. QRT-PCR showed that overexpression of KPNA2 enhanced the expressions of PFK2, PKM1. On the contrary, KPNA2-knockdown cells showed the opposite effects (Fig. 2b, Additional file 1: Figure S1B). Although there was no dramatic change associated with the mRNA of HK2, the enzymatic activities of all three enzymes were significantly enhanced when KPNA2 was up-regulated and decreased when KPNA2 was depleted (Fig. 2c, Additional file 1: Figure S1C). These data evidently suggested that KPNA2 might play an important role in promoting glycolysis of the glioma cells.

Lactic acid production is one of the key features of Warburg effect. We therefor measured the level of lactic acid in the culture medium conditioned by U87 and U251 cells. As the results shown in Fig. 2d, the lactate level in the culture medium was notably increased when KPNA2 was overexpressed and significantly decreased when KPNA2 was knockdown, consistent with the results of the glycolytic enzymes (Fig. 2d, Additional file 1: Figure S1D). To further investigate the lactate metabolism in these cells, we measured the expressions of MCT1 and MCT4 by q-PCR, which were responsible for lactate transport. The results showed that MCT1 was decreased in the KPNA2-knockdown cells, and increased when KPNA2 was overexpressed. In contrast, MCT4 showed differential expressions in the U87 and U251 cells. Specifically, MCT4 was decreased in the U87 cells and increased in the U251 cells respectively when KPNA2 was depleted (Fig. 2e, Additional file 1: Figure S1E).

Glucose uptake is an important biological process of energetic metabolism, especially in cancer cells. 2-deoxyglucose (2-DG) has been widely used because of its structural similarity to glucose. We tested the uptake of 2-DG by colorimetric assay. The results showed that overexpression of KPNA2 led to more absorption of 2-DG than the control cells (Fig. 2f, Additional file 1: Figure S1F). Additionally, we measured the plasma membrane expression of glucose transporter 1 (GLUT-1),which plays a key role in extracellular deoxyglucose uptake. As the data shown in Fig. 2e, in comparison to the control cells, GLUT-1 residing on the plasma membrane was substantially up-regulated in the cells overexpressed with KPNA2 (Fig. 2g, Additional file 1: Figure S1G). On the contrary, KPNA2-depleted cells showed lower levels of both GLUT-1 and 2-DG uptake (Fig. 2f, g; Additional file 1: Figure S1F, G).

### KPNA2 regulated the mitochondrial respiration and cell proliferation in the glioblastoma cells

**Fig. 3:**
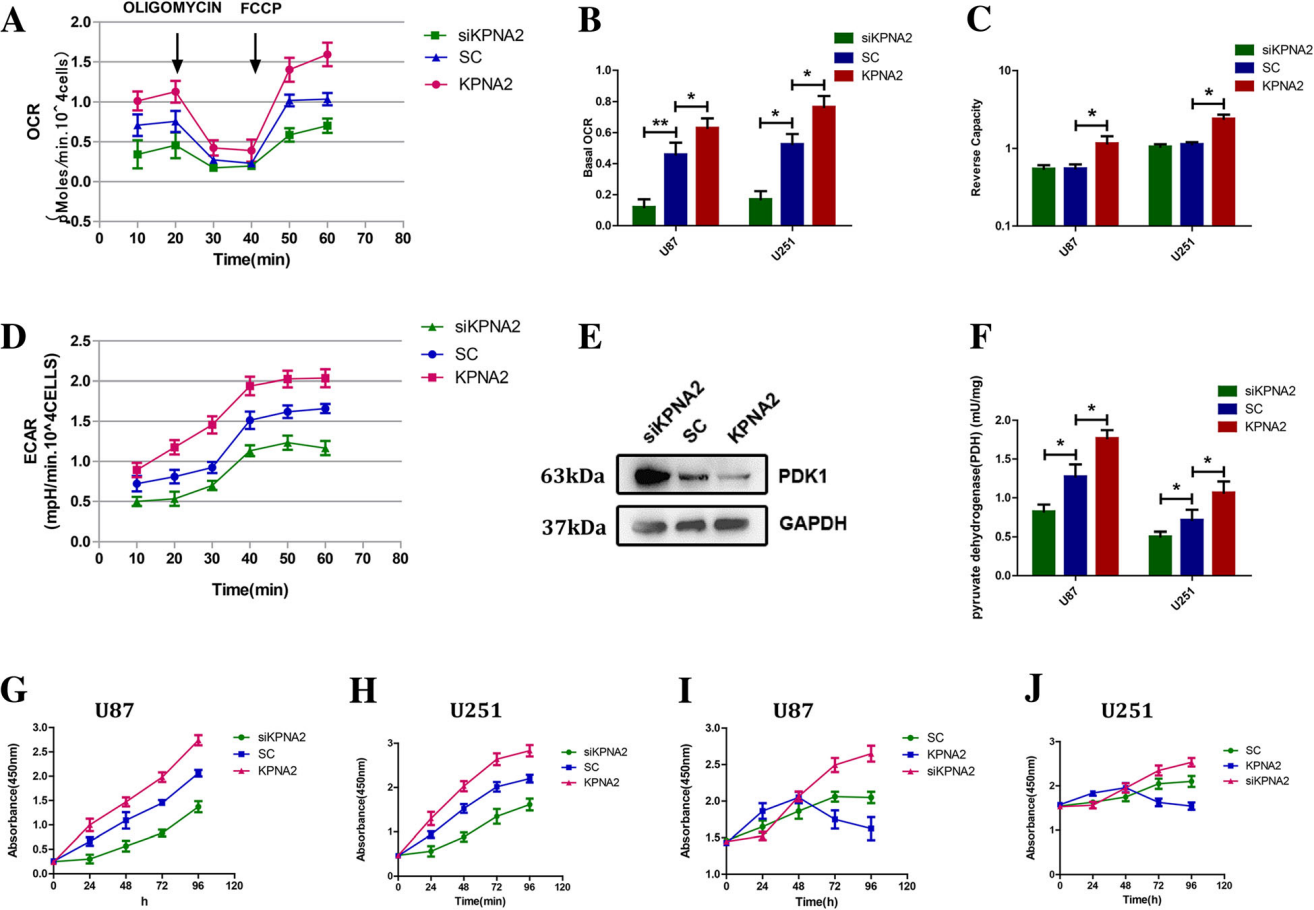
OXPHOS and cell growth were regulated by KPNA2 in the glioblastoma cells. **a** OCRs were determined in the U87 glioblastoma cells transfected with SC-shRNA, KPNA2-shRNA or wild-type KPNA2. Oligomycin (1 mM) and FCCP (300 mM) were sequentially added at 20th minutes and 40th minutes. **b** Basal OCR (original OCR minus OCR after addition of oligomycin and **c** reserve capacity (maximal OCR induced by minus basal OCR) were calculated. Results were presented as the mean ± s.d. from three independent experiments. *P < 0.05, **P < 0.01. **d** ECARs in the U87 cells were detected using the same methodology with OCRs. **e** Expressions of PDK1 were detected by immunoblotting in the U87 cells transfected with SC-shRNA, KPNA2-shRNA or wild-type KPNA2. **f** Enzymatic activities of PDH were determined in the U87 and U251cells transfected with KPNA2-shRNA, scrambled shRNA and wild-type KPNA2. Growth curves of the transduced cells were shown, cultured in the (**g**—U87, **h**—U251) glucose-containing (4500 mg/l) or (**i**—U87, **j**—251) glucose-free (w/o GLU) medium. Each bar represented the mean ± s.d. from three independent experiments

To examine the possible effects of KPNA2 on the oxidative phosphorylation (OXPHOS), we added oligomycin, a pharmacological inhibitor of electron transport, followed by FCCP, a mitochondrial uncoupler, to the U87 and U251 cells (Fig. 3a, Additional file 1: Figure S2A) and tested their oxygen consumption rates (OCRs). The results showed a decrease followed by an increase in the levels of OCRs in all the cell groups. Moreover, both the basal OCR and the calculated reserve capacity were decreased in the cells knock-down of KPNA2, and moderately increased in the KPNA2 up-regulated cells (Fig. 3b, c). On the other hand, the extracellular acidification rate (ECAR) was reduced in the KPNA2-depleted cells and elevated when KPNA2 was overexpressed, unsurprisingly consistent with the lactate production data in Fig. 2d(Fig. 3d, Additional file 1: Figure S2B).

Pyruvate dehydrogenase (PDH), an important factor in the OXPHOS, stands at the crossroad of glycolysis and mitochondrial respiration. We first examined the expression of pyruvate dehydrogenase kinase 1 (PDK1), which phophorylates and inactivates pyruvate dehydrogenase (PDH). The results showed that the mRNA level of PDK1 was decreased in the KPNA2-overexpressed cells and increased in the cells knock-down of KPNA2 (Fig. 3e, Additional file 1: Figure S2C). We subsequently measured the enzymatic activity of PDH. Indeed, it showed significant reduction in the KPNA2-knockdown cells and concomitant increase in the cells overexpressed with KPNA2 (Fig. 3f).

Since cell growth is directly linked to the balance between oxidative and glycolytic metabolism, we next examined the growth kinetics of the U87 and U251 cells overexpressed or depleted of KPNA2. In comparison to the controls, the cells up-regulated with KPNA2 exhibited significantly increased growth kinetics, indicating a positive role of KPNA2 in cell proliferation (Fig. 3g, h). When we cultured the cells into the glucose-free medium, the growth rate of these cells slowed down dramatically (Fig. 3i, j). On the contrary, level of glucose had a minor effect on the growth kinetics of the glioma cells when KPNA2 was depleted, suggesting that the cells with higher level of KPNA2 relied on glucose as their major energy source.

To further investigate whether the cells with low or no expression of KPNA2 survived glucose restriction most likely through their capacity to metabolize other nutrients, we have investigated the expressions of GLS and SLC1A5, two key factors involved in glutamine uptake and catabolism. The results showed that both SLC1A5 and GLS were indeed increased in the U251 cells when KPNA2 was down-regulated and decreased when KPNA2 was overexpressed, evidently suggesting that KPNA2 might have multiple effects on the metabolic reprogramming to sustain intracellular stability (Additional file 1: Figure S2D).

### Targeting KPNA2 in the glioblastoma cells decreased tumorigenic capacity and increases survival of mice bearing xenografts

**Fig. 4:**
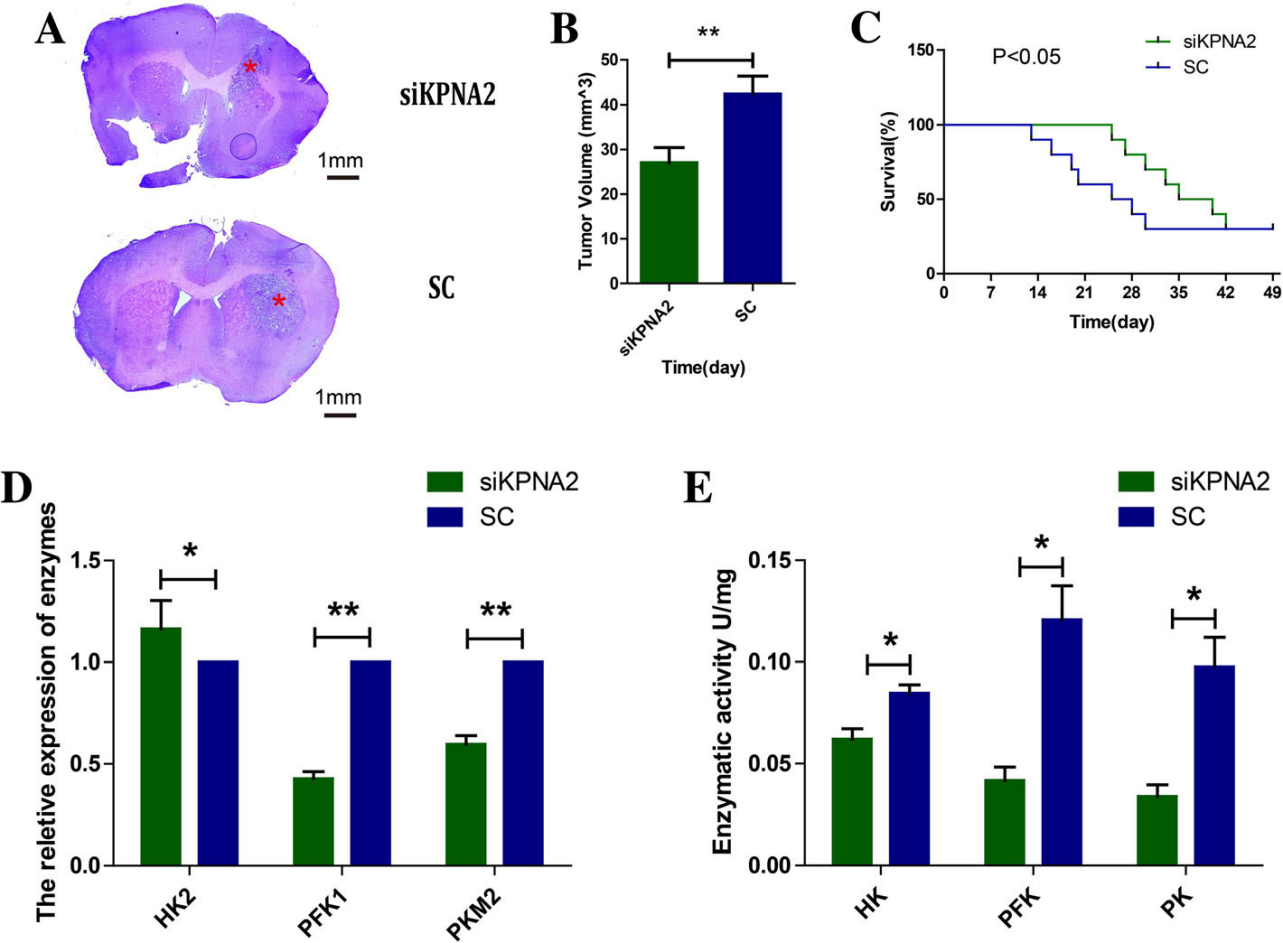
KPNA2 was associated with the metabolic reprogramming in the glioma xenografts. The U87 cells transfected with small hairpin RNAs of KPNA2 or scrambled nonspecific shRNAs were implanted into the right frontal lobes of the immunocompromised mice. **a** Representative H&E staining images of mouse brains at 42 days after inoculation were shown. Asterisk indicates tumor (scale bar, 1 mm) **b** The estimated tumor volumes at that time. Data were presented as the mean ± s.d. from three independent experiments. **P < 0.05. **c** Curve graph indicated survival time of the xenografted mice. *P* < 0.05 is considered statistically significant. **d** The relative expressions and **e** the activities of key enzymes involved in the glycolytic metabolism of the xenograft tumors were determined. Data were presented as the mean ± s.d. from three independent experiments.*P < 0.05, **P < 0.01

Considering the in vitro involvement of KPNA2 in glioma cell proliferation, survival and metabolism, we extended our study to determine the impact of KPNA2 depletion on tumorigenic capacity of glioma cells in vivo. When the KPNA2-knockdown U87 cells implanted into the right frontal lobes of immunocompromised mice, apparent decrease in tumor formation (Fig. 4a, b) and subsequent increase in the survival time of the tumor-bearing mice were observed (Fig. 4c).

In addition, we tested the mRNA levels of HK2, PFK1 and PKM2 in the xenograft tumors by qRT-PCR analysis. The results showed that except for HK2, depletion of KPNA2 resulted in dramatically lower levels of the respective enzyme (Fig. 4d). Their enzymatic activates were decreased as well (Fig. 4e), indicating that KPNA2-knockdown xenograft tumors had lower level of glycolytic metabolism. In a word, KPNA2 might play an important role in the metabolic transformation of glioblastoma cells, which exerts vital effects on tumor initiation and progression.

### C-myc mediated the role of KPNA2 in regulating the glycolytic metabolism of glioma cells

C-myc is one of major oncogenic factors involved in different human cancers. It acts as a transcriptional factor to activate genes that permit metabolic adaptation, such as HK2, PFK1, PKM2 and GLUT1. Considering the broad involvement of KPNA2 in the metabolic reprogramming and tumor growth of glioblastomas, we hypothesized that the role of KPNA2 in glioblastomas may be linked with c-myc.

**Fig. 5:**
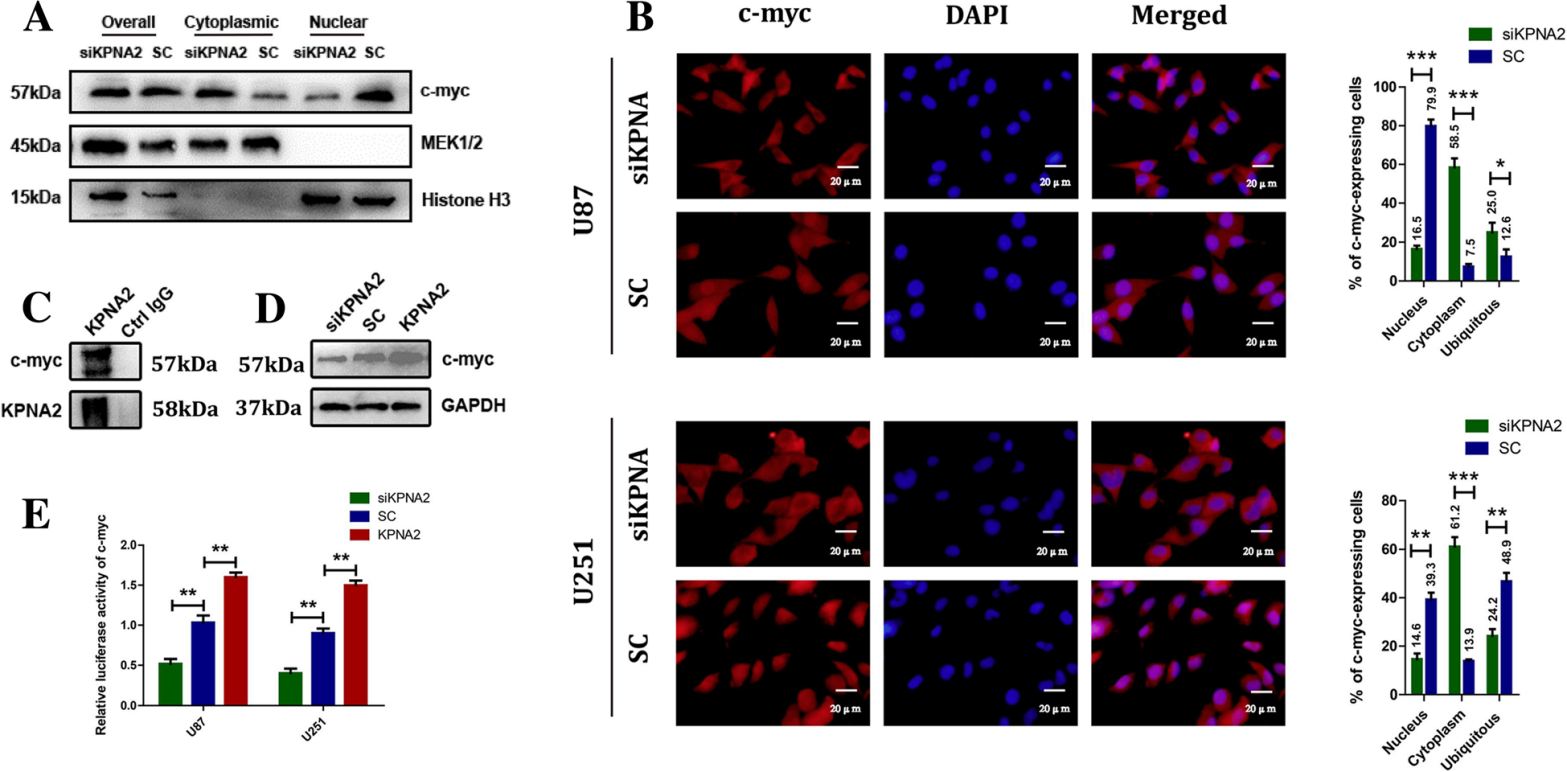
KPNA2 mediated both the translocation and the transcriptional expression of c-myc. The subcellular distribution of c-myc in the KPNA2-shRNA and control transfected cells by nuclear/cytosol fractionation (**a**) and immunofluorescence (**b**) was shown. Error bars indicated the mean ± s.d. of three independent views. MEK1/2 was used as the cytoplasmic control and Histone H3 as the nuclear control. *P < 0.05, ***P < 0.001. **c** Co-immunoprecipitation analysis was presented between KPNA2 and c-myc in the U87 cells. **d** Expressions of c-myc in the U87 cells knockdown or overexpressed of KPNA2 were determined by immunoblotting. **e** The U87 and U251 cells were transfected with pGL2-c-myc, a vector encoding KPNA2-shRNA, wild-type KPNA2 or its comparative control, and pSV-Kerilla. Values in graphs represented the mean of Fluc:Rluc activity±s.d. performed in triplicate. **P < 0.01

To verify this hypothesis, we first measured the subcellular localization of c-myc in the KPNA2 depleted or overexpressed cells. Both nuclear/cytosol fractionation and immunofluorescence (IF) analyses (Fig. 5a, b) indicated that more c-myc was retained in the cytoplasm when KPNA2 was depleted. Additionally, co-immunoprecipitation (CO-IP) analysis demonstrated that KPNA2 interacted directly with c-myc in the U87 cells (Fig. 5c). A comparable result was observed in the U251 glioblastoma cells (data not shown), further suggesting that KPNA2 played an important role in mediating the transportation of c-myc into the nucleus.

We additionally examined the expressions of c-myc in these cells. Interestingly, western bolt analysis indicated that protein level of c-myc was decreased in the cells knockdown of KPNA2 and increased in the cells when KPNA2 was overexpressed (Fig. 5d). When we introduced a luciferase reporter into these cells, a significant increase in the luciferase activity was coupled with up-regulation of KPNA2, whereas knockdown of KPNA2 in the U87 and U251 cells reduced c-myc transcriptional activity (Fig. 5e). These results evidently suggested that KPNA2 might play dual effects on both translocation as well as expression of c-myc.

### KPNA2 upregulated the transcriptional expression of c-myc by mediating the translocation of E2F1 into the nucleus

**Fig. 6:**
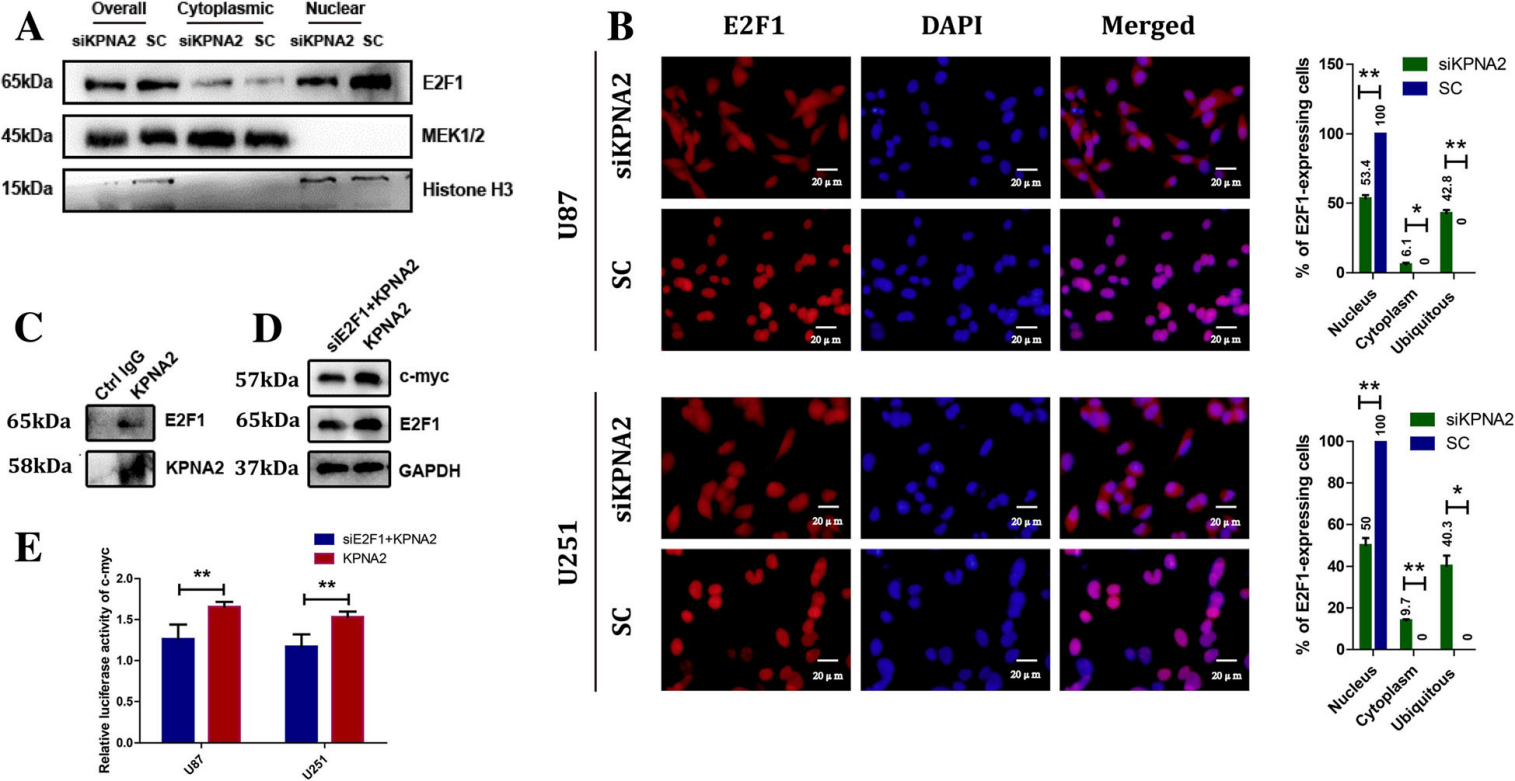
Subcellular redistribution of E2F1 caused by knockdown of KPNA2 contributed to the deregulation of c-myc. The subcellular distribution of E2F1 in the KPNA2-shRNA and control transfected cells by nuclear/cytosol fractionation (**a**) and immunofluorescence (**b**) was shown. Error bars indicated the mean ± s.d. of three independent views. MEK1/2 was used as the cytoplasmic control and Histone H3 as the nuclear control. *P < 0.05, **P < 0.01. **c** Co-immunoprecipitation analysis was presented between KPNA2 and E2F1 in the U87 cells. **d** Expressions of c-myc were analyzed by immunoblotting in the KPNA2-overexpressed U87 cells silenced with E2F1 or not. **e** The KPNA2-overexpressed U87 and U251 cells were transfected with pGL2-c-myc, a vector encoding E2F1-shRNA or its comparative control, and pSV-Kerilla. Values in graphs represented the mean of Fluc:Rluc activity±s.d. performed in triplicate. **P < 0.01

In consideration of KPNA2 role in c-myc transactivation, we applied ChIP assay to the glioma cells. However, no KPNA2 was detected on the promoter region of c-myc compared to the negative controls (data not shown). We therefore speculated that there may be other pathways/factors that mediated the transcriptional regulation of c-myc by KPNA2. E2F1, another cargo protein of KPNA2 [24], is a key factor that participates in regulating cell cycle [25], proliferation [26], apoptosis [27], senescence [28], and DNA-damage response [29]. It has been reported that E2F1 up-regulated c-myc on the transcriptional level. On the other hand, pRb down-regulated c-myc by sequestering E2F1 [30, 31]. In view of this, we hypothesized that in glioma cells, E2F1 may be involved in the transcriptional regulation of c-myc by KPNA2. To test this hypothesis, we first examined the effects of KPNA2 exerting to E2F1 in the glioma cells. The results showed translocation of E2F1 was significantly decreased when KPNA2 was knockdown by both the nuclear/cytosol fractionation and immunofluorescence analyses (Fig. 6a, b). Direct interaction between KPNA2 and E2F1 was further confirmed by the CO-IP assay (Fig. 6c). We subsequently transfected E2F1-shRNA vector into the U87 cells overexpressed with KPNA2. The results showed that the originally up-regulated c-myc on the levels of both mRNA and luciferase activity was significantly decreased when E2F1 was depleted (Fig. 6d, e).

### Knockdown of c-myc partially reversed the metabolic transformation caused by KPNA2

**Fig. 7:**
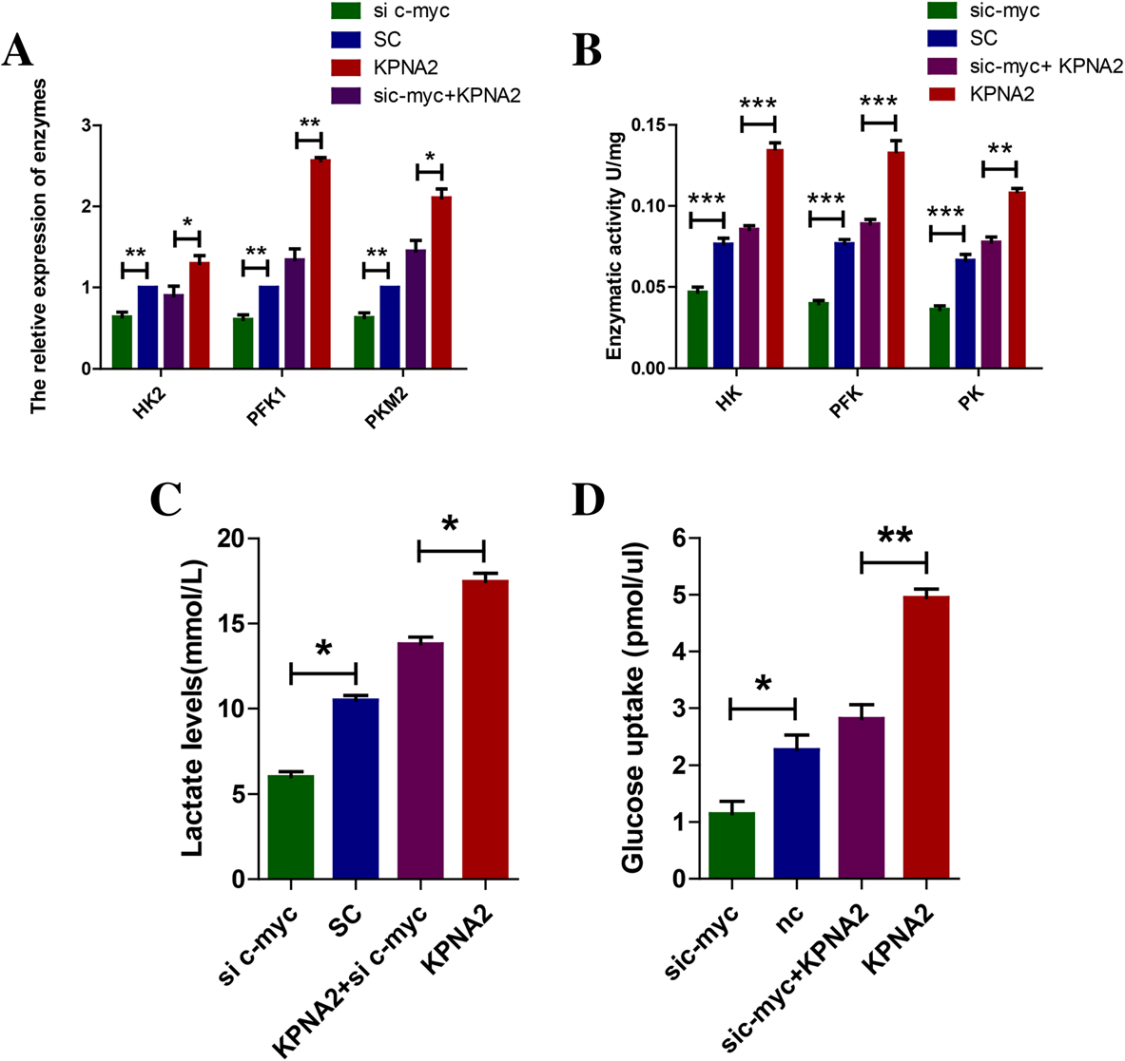
Knockdown of c-myc partially reversed the glycolytic reprogramming caused by overexpression of KPNA2. **a** mRNA levels were determined in the U87 cells transfected with c-myc-shRNA and scrambled shRNA. Each bar represented the mean ± s.d. from three independent experiments. **P < 0.01. **b** Enzymatic activities of HK, PKM and PFK1 in the U87 cells transfected with c-myc-shRNA, scrambled shRNA, c-myc-shRNA+ wild-type KPNA2, wild-type KPNA2 were examined. Each bar represented the mean ± s.d. from three independent experiments. **P < 0.01, ***P < 0.001. **c** Lactate production was measured in the indicated cells. Data were presented as the mean ± s.d. from three independent experiments. *P < 0.05. **e** Relative deoxyglucose uptake was measured in the indicated cells. Each bar represented the mean ± s.d. from three independent experiments. *P < 0.05, ** *P* < 0.01

To confirm the metabolic effects of KPNA2 in the glioma cells was dependent on c-myc, we transfected the shRNA vector targeting c-myc into the U87 cells either overexpressed with KPNA2 or its comparative control. The results showed that depletion of c-myc significantly decreased the mRNA levels of PKM2, PFK1 and HK2 (Fig. 7a, Additional file 1: Figure S3A). Similar results were observed in terms of the enzymatic activities of PKM, PFK and HK as well as production of lactic acid (Fig. 7b, c, Additional file 1: Figure S3B, C). Glucose uptake in the KPNA2 up-regulated cells concomitantly decreased when c-myc was knockdown (Fig. 7d, Additional file 1: Figure S3D), further suggesting that c-myc was indeed a target protein that was involved in regulation of glycolytic metabolism and tumorigenesis by KPNA2.

## Discussion

It has been proposed that energy metabolism reprogramming is a novel hallmark of cancer cells [32]. The switch from oxidative respiration to glycolysis provides an acidic microenvironment that facilitates tumor invasion and suppresses anti-cancer T cell immune responses [33]. Decreased production of ROS in accordance to lower cellular respiration may protect cancer cells from apoptosis [34]. In tumor tissues, cells with different metabolic states formed intra-tumoral heterogeneity, which is responsible for the failure to induce the same therapeutic effect against cancer cells [35]. Moreover, the intermediates such as nucleosides and amino acids generated by increased glycolysis participate into various biosynthetic pathways, and facilitate biosynthesis of the macromolecules and organelles required for assembling new cells. Indeed, this “Warburg-like” metabolism has often been observed in many rapidly dividing embryonic tissues, further suggesting its crucial role in active cell proliferation [36]. Therefore, a very important technique called F18-fluorodeoxyglucose positron emission tomography (PET) has been developed and widely used in clinics based on this metabolic feature for diagnosis of malignant cells. Arising from present results, our study on KPNA2 expanded the views on the mechanisms for Warburg effect, which would provide a new perspective on the diagnosis and treatment of cancers.

Overexpression of KPNA2 has been commonly observed in many kinds of cancers, even in precancerous lesions, indicating that KPNA2 is a crucial element for cancer occurrence, development and prognosis. Due to its function in nucleocytoplasmic transport, KPNA2 participates in many cellular processes and thus provides an important therapeutic target aiming at tumors. Previous studies showed that KPNA2 was mainly related to cell circle, and DNA metabolic processes [24]. For example, P53 is a multiple functional factor that is involved in cell proliferation, apoptosis and autophagy. Newly studies revealed that some conventional drugs used to treat diseases other than cancers can have antitumor therapeutic effects. For example, capsaicin, can induce autophagy by restoring wild-type p53 activities over mutant p53 functions [37]. KPNA2 mediates the nuclear transport of P53. When the nuclear translocation of P53 in the glioblastoma cells was deregulated by KPNA2 disturbance, cancer migration, infiltration and autophagy were significantly inhibited. Therefore, induction of autophagy through KPNA2-P53 pathway may be a new therapeutic strategy for cancers [38].

In this study, we performed IHC analysis and confirmed that KPNA2 was indeed elevated in the glioma specimens compared to the normal brain tissues. High level of KPNA2 predicted poor prognosis for the glioma patients. Furthermore, down-regulation of KPNA2 in the glioblastoma cells caused a remarkable decrease of cell viability and rise in the survival rates of xenograft mice, evidently supporting the association between high level of KPNA2 and a worse clinical outcome. In spite of the crucial roles were disclosed, the mechanisms of KPNA2 in cancers, especially on cancerous metabolism still remains to be elucidated.

**Fig. 8:**
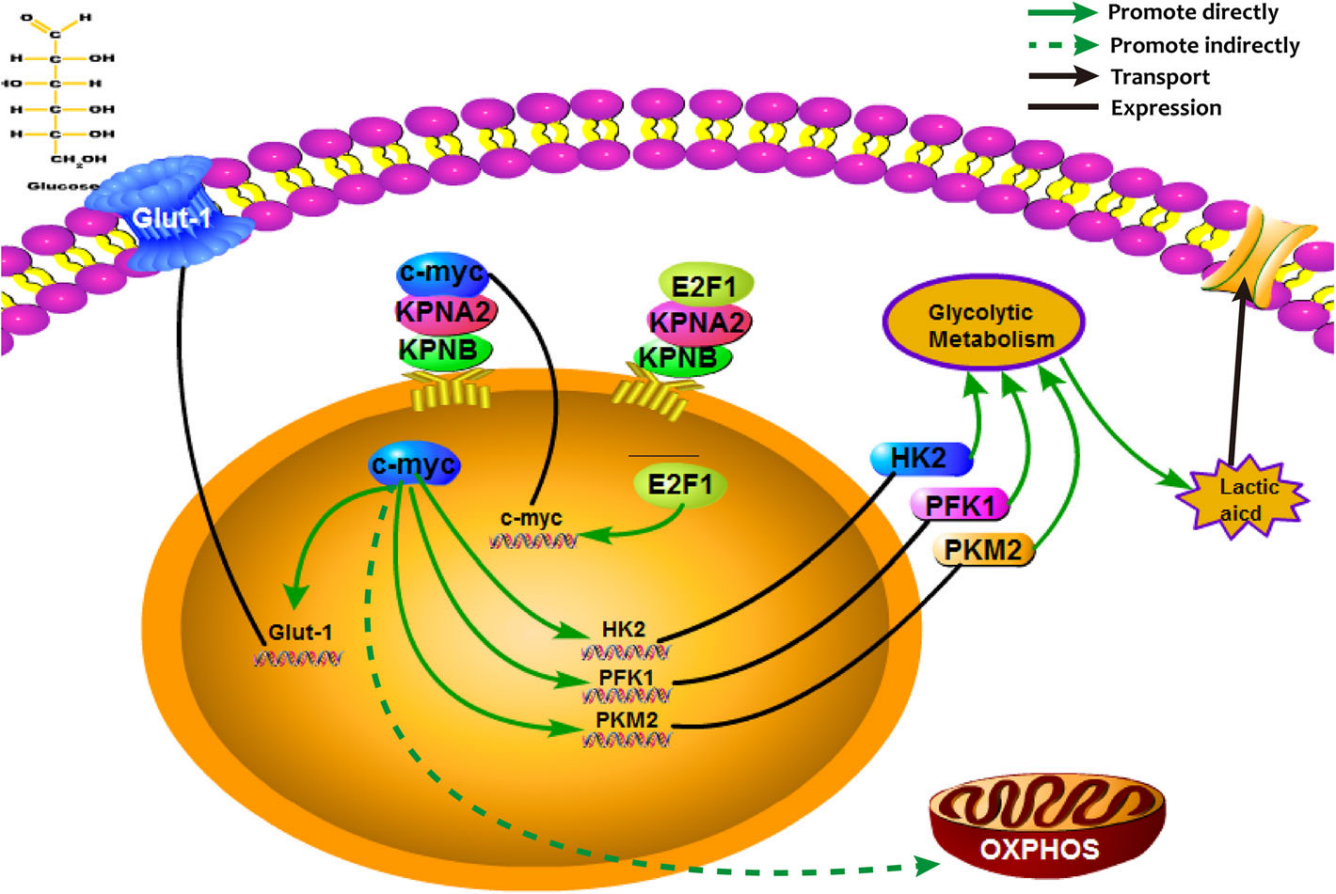
A mechanistic diagram was shown indicating how KPNA2 was involved in the metabolic reprogramming of glioma cells

As shown in Fig. 8, we observed that glycolytic metabolism such as activities of the key enzymes in glycolysis and lactic acid production was significant decreased in the KPNA2-knockdown glioma cell lines U87 and U251. Down-regulation of Glut-1, as an important feature of decreased glucose uptake was also shown [39–41]. Except for the traditional Warburg effect, the importance of mitochondrial metabolism in cancers has been widely concerned. As shown in Fig. 3, depletion of KPNA2 resulted in a modest decrease in the mitochondrial aerobic respiration. Growth kinetics of the cells was additionally suppressed when KPNA2 was knockdown, evidently indicating that KPNA2 might have multiple effects on the metabolic reprogramming of gliomas.

HIF family proteins and c-myc play crucial roles in glycolytic metabolism and promote metabolic heterogeneity and plasticity under different environments [42]. HIF1-α and HIF2-α, which are activated under hypoxic conditions, promote the glycolytic metabolism and suppress the activities of mitochondria. Additionally they play crucial roles in activating the stem-like properties of cancer cells. For example, HIF2-alpha was recently shown to contribute in a cooperative manner with the intracellular domain of CD44 to the acquisition of radio-resistance by glioma stem cells in a perivascular niche [43]. Expression of CD44, which correlates with cell proliferation and phenotype stability, on the other hand, promotes the metabolic heterogeneity and plasticity of the gliomas [44]. C-myc could concurrently drive aerobic glycolysis and/or oxidative phosphorylation to provide sufficient energy and anabolic substrates for cell growth and proliferation in the context of the tumor microenvironment [45]. C-myc is a powerful oncogene, which is regulated by many elements. For example, ROS-induced canonical Wnt/beta-catenin signal pathway transcriptionally activates c-myc, thus accelerates cell proliferation and oncogenicity [46]. CD44, one of the cancer stem cell markers, is activated as well. In contrast, ESRP1-CD44 variant 8-10-xCT cystine transporter axis acts as a negative feedback to protect cells from redox stress. In that case, c-myc is no longer activated transcriptionally [47, 48]. We have previously tested that HIF1α showed no obvious changes in the KPNA2-depleted or overexpressed cells (data not shown), and therefore focused on c-myc as a target of KPNA2. As expected, various analyses such as nuclear/cytosol fractionation, IF and CO-IP indicated that KPNA2 regulated the functions of c-myc by mediating its translocation into the nucleus. Interestingly, we additionally discovered that transactivation of c-myc was affected by KPNA2 as well. Ruling out the direct binding between KPNA2 and the promoter region of c-myc, we were searching for possible transcriptional factors targeted on c-myc. As such, E2F1, another cargo protein of KPNA2, attracted our attention. E2F1 is a multi-functional factor involved in many biological processes. Not only sharing similar biological functions, E2F1 has complex interactions with c-myc. For instance, E2F1 directly binds to the P2 promoter region of c-myc and facilitates its transcriptional activation [30]. C-myc, on the other hand regulates E2F1 on both levels of transcription and post-transcription mediated by members of miR-17-92 cluster. Nowadays, more and more studies have linked E2F1 with the metabolic reprogramming in cancer cells [49–51]. It was reported that E2F1 played a key role in regulation of glycolysis in sirt6 mediated pathway. What’s more, up-regulation of PDK1 and concomitant reduction in the activity of PDH was demonstrated in the cells depleted of E2F1. In view of this, we focused our attention on investigating the interactions between KPNA2, c-myc and E2F1 in the glycolytic transformation of gliomas. Our results showed that besides c-myc itself, KPNA2 mediated translocation of E2F1 into the nucleus, where E2F1 regulated the expression of c-myc on the transcriptional level directly. As such, KPNA2 acted as a potential oncogenic factor by double targeting the translocation as well as expression of c-myc.

In summary, we have provided unequivocal evidence demonstrating the KPNA2 performs its function in part via regulating cellular metabolism through c-myc. The novel finding would provide a new perspective to roles of KPNA2 in tumorigenesis and progression. As a corollary, regulation of tumor metabolism by KPNA2 may provide an important area for cancer diagnosis and therapeutic intervention.

## Conclusions

Our findings elucidated that KPNA2, a potential tumor oncogenic protein, performs its function in part via regulating cellular metabolism through c-myc signaling axis. It would provide a possible explanation for Warburg effect and thus offer a new perspective to the roles of KPNA2 in gliomagenesis.

## Additional file

Additional file 1: **Figure S1.** KPNA2 promoted the glycolytic metabolism in the U251 glioblastoma cells. **Figure S2.** KPNA2 affects the OXPHOS and Glutaminolysis in the glioma cells. **Figure S3.** Knockdown of c-myc partially reversed the glycolytic reprogramming caused by overexpression of KPNA2. (DOCX 526 kb)

## Abbreviations

2-DG: 2-deoxyglucose
c-myc: MYC proto-oncogene
E2F1: E2F transcription factor 1
ECAR: Extracellular acidification
ECAR: Extracellular acidification rate
GBM: Glioblastoma multiforme
GLUT1: Glucose transporter 1
HK: Hexokinase
IHC: Immunohistochemistry
KPNA2: Karyopherinα2
OCR: Oxygen consumption rates
OXPHOS: Oxidative phosphorylation
PDH: Pyruvate dehydrogenase
PDK1: Pyruvate dehydrogenase kinase 1
PFK: Phosphofructokinase
PKM: Pyruvate kinase
qRT-PCR: Quantitative real-time PCR
ROS: Reactive oxygen species
