# Supplementary material for: KPNA2 promotes metabolic reprogramming in glioblastomas by regulation of c-myc
Source: J Exp Clin Cancer Res. 2018 Aug 16;37:194. doi: 10.1186/s13046-018-0861-9 (PMC6097452; doi:10.1186/s13046-018-0861-9)
Supplement: Supplementary file 1 — Figure S1. KPNA2 promoted the glycolytic metabolism in the U251 glioblastoma cells. Figure S2. KPNA2 affects the OXPHOS and Glutaminolysis in the glioma cells. Figure S3. Knockdown of c-myc partially reversed the glycolytic reprogramming caused by overexpression of KPNA2. (DOCX 526 kb) [file 13046_2018_861_MOESM1_ESM.docx]

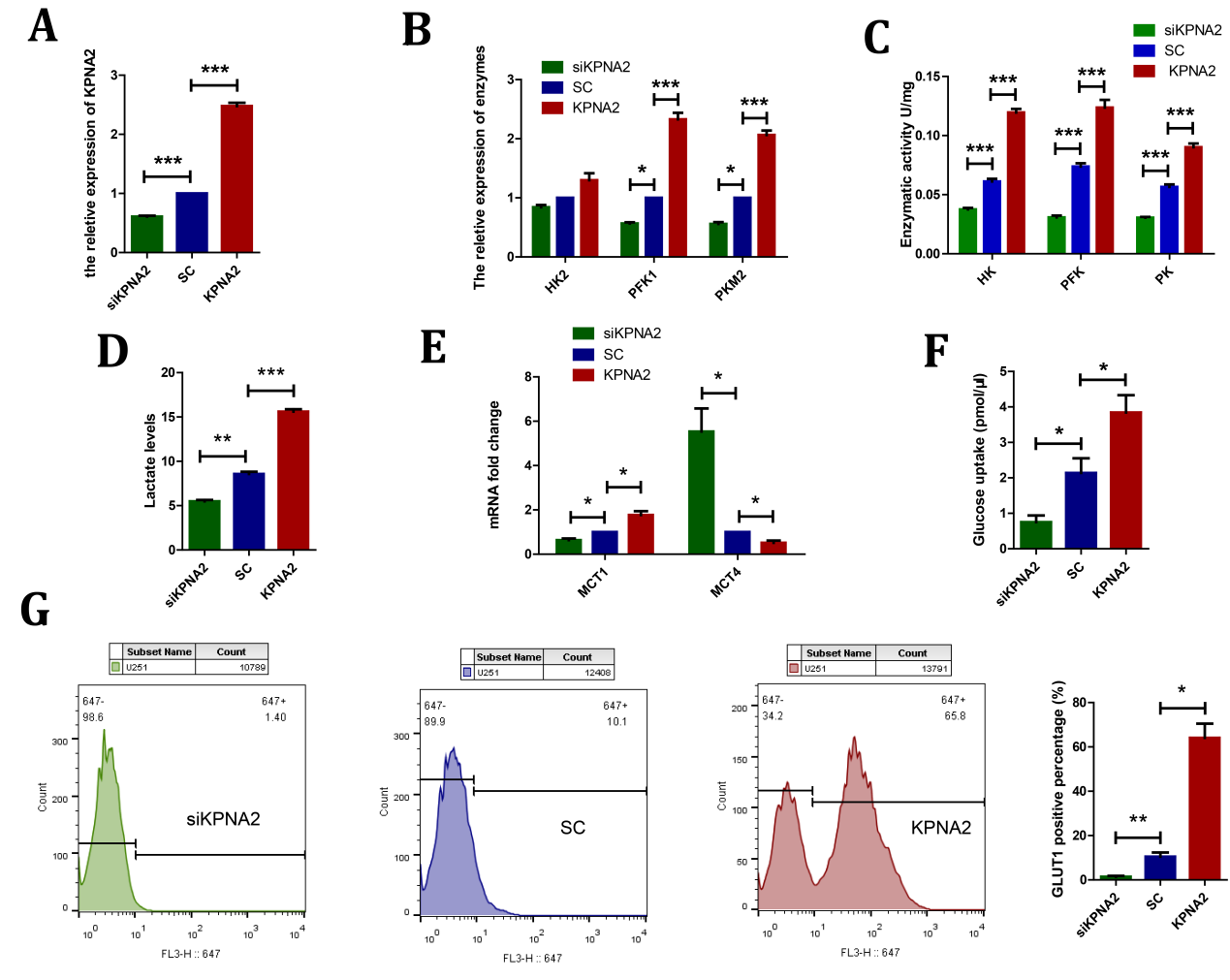


**Figure S1: KPNA2 promoted the glycolytic metabolism in the U251 glioblastoma cells**

(A) Levels of KPNA2 mRNA were analyzed by qRT-PCR in the U251 glioblastoma cells transfected with the lentiviruses expressing small hairpin RNA of KPNA2(shKPNA2), wild-type KPNA2 (KPNA2), or a vector with scrambled nonspecific shRNAs(SC). GAPDH served as a loading control. (B) mRNA levels and (C)enzymatic activities of HK2, PKM2 and PFK1 were determined in the U251 cells transfected with KPNA2-shRNA, scrambled shRNA and wild-type KPNA2. Each bar represented the mean ± s.d. from three independent experiments. *P < 0.05, ***P < 0.001. (D) Lactate production was measured in the indicated cells. Data were presented as the mean ± s.d. from three independent experiments. **P < 0.01. (E) MRNA levels of MCT1 and MCT4 in the indicated U251 cells. Data were presented as the mean ± s.d. from three independent experiments. *P < 0.05. (F) Relative deoxyglucose uptake was measured in the indicated cells. Each bar represented the mean ± s.d. from three independent experiments. ***P < 0.001. (G) The expression of GLUT-1 was detected by flow cytometry in the indicated cells, cells were marked with Alexa Flour®647-GLUT1 antibody and the statistic analyze were followed. Data were presented as the mean ± s.d. from three independent experiments. *P < 0.05, **P < 0.01.


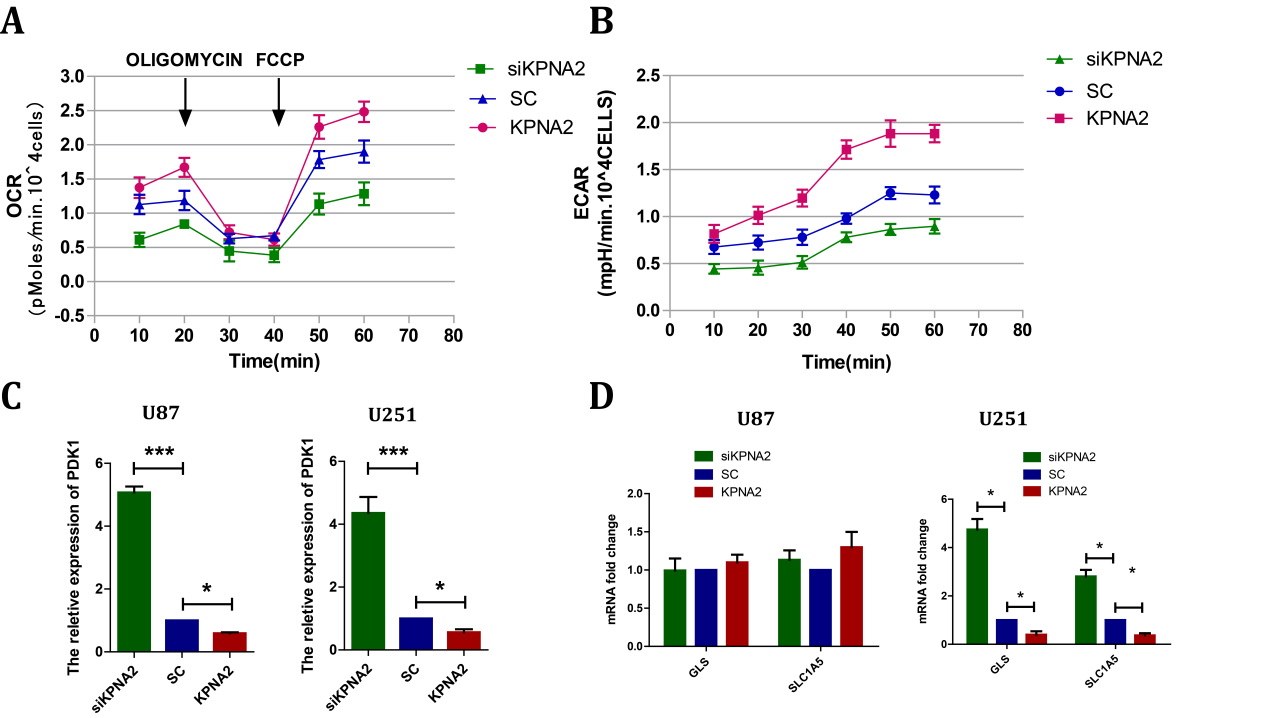


**Figure S2: KPNA2 affects the OXPHOS and Glutaminolysis in the glioma cells.**

(A) OCRs and (B) ECARs were determined in the U251 glioblastoma cells transfected with SC-shRNA, KPNA2-shRNA or wild-type KPNA2. Each bar represented the mean ± standard from three independent experiments. (C) The mRNA level of PDK1 was determined in the U87 and U251 cells transfected with KPNA2-shRNA, scrambled shRNA or wild-type KPNA2. Each bar represented the mean ± s.d. from three independent experiments. *P < 0.05, ***P < 0.001 (D) The expression of GLS and SLC1A5 expression in the indicated cells.


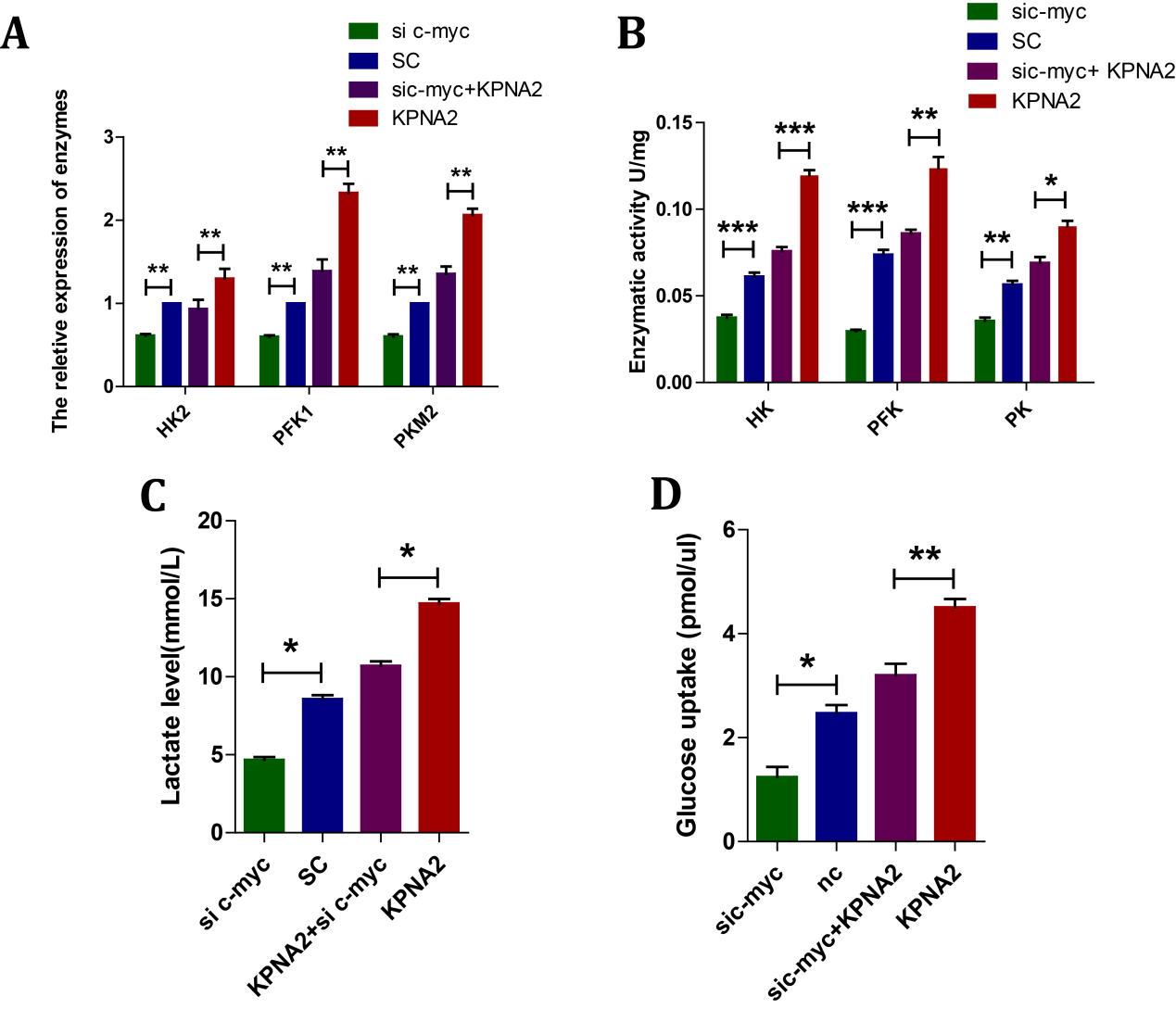


**Figure S3: Knockdown of c-myc partially reversed the glycolytic reprogramming caused by overexpression of KPNA2**

(A) mRNA levels were determined in the U251 cells transfected with c-myc-shRNA and scrambled shRNA. Each bar represented the mean ± s.d. from three independent experiments. **P < 0.01. (B) Enzymatic activities of HK, PKM and PFK1 were examined in the U251 cells transfected with c-myc-shRNA, scrambled shRNA, c-myc-shRNA+ wild-type KPNA2, wild-type KPNA2. Each bar represented the mean ± s.d. from three independent experiments. *P<0.05, **P < 0.01, ***P<0.001. (C) Lactate production was measured in the indicated cells. Data were presented as the mean ± s.d. from three independent experiments. *P < 0.05. (E) Relative deoxyglucose uptake was measured in the indicated cells. Each bar represented the mean ± s.d. from three independent experiments. *P < 0.05, ** P<0.01.
